# Understanding the Role of Vitamin D in Heart Failure

**DOI:** 10.31083/j.rcm2404111

**Published:** 2023-04-17

**Authors:** Paramjit S. Tappia, Rhea Lopez, Shirley Fitzpatrick-Wong, Bram Ramjiawan

**Affiliations:** ^1^Asper Clinical Research Institute & Albrechtsen Research Centre, St. Boniface Hospital, Winnipeg, MB R2H 2A6, Canada; ^2^Department of Pharmacology and Therapeutics, Rady Faculty of Health Sciences, Max Rady College of Medicine, University of Manitoba, Winnipeg, MB R3E 0T6, Canada

**Keywords:** vitamin D, heart failure, nutritional interventions, preventive nutrition

## Abstract

Vitamin D is now believed to have a significant role in cardiac signal transduction and regulation of gene expression, and thus influences normal cardiomyocyte function. It has been reported to provide cardioprotection through its anti-inflammatory, anti-apoptotic and anti-fibrotic actions; and to prevent cardiac remodeling, ${\mathrm{Ca}}^{2+}$-handling defects, and abnormal electrophysiological patterns. A vitamin D deficient state has been associated in the pathogenesis of heart failure; however, while many clinical studies report a benefit of vitamin D to heart function, other clinical studies are inconsistent with these findings. These uncertainties have led to a discord in the recommendation of vitamin D supplementation for the treatment of heart failure or as a preventive agent in patients deemed to be at risk for cardiac dysfunction. Accordingly, this article is intended to describe some of the mechanisms/sites of action of vitamin D in different animal models of heart failure, as well as to review the clinical observations and challenges in the interpretation and understanding of the clinical relevance of vitamin D in relation to heart function.

## 1. Introduction

Emerging data is suggestive that vitamin D may influence cardiac structural and contractile function and that vitamin D status may influence heart failure patient outcomes. In fact, insufficient vitamin D levels have been observed in patients with heart failure [1, 2]. Indeed, it is now generally believed that a deficiency of vitamin D is one of the most frequently observed and reported pathophysiological condition and as a consequence has become a major global public health concern. With respect to the 2005 and 2006 National Health and Nutrition Examination Survey (NHANES) in the U.S., approximately 42% of the study participants were deemed to exhibit insufficient levels of vitamin D. Subsequent NHANES data between 2011–2012, reported a deficiency of serum vitamin D concentration (<50 nmol/L) in almost 40% of the study population [3].

In the U.S., Canada, Europe, Australia, New Zealand, and Asia it has been proposed that one third to a half of the children and adult population are in a state of vitamin D deficiency. In spite of the the importance of skin exposure to sunlight for the sytnthesis of vitamin D, high incidence of vitamin D deficiency have been reported in the geographical sunniest regions, such as the Middle East and South Asia that have been attributed to low exposure to sunlight related cultural elements [4]. While epidemiological and accumulating experimental lines of evidence demonstrate a link between vitamin D deficiency and the incidence of heart failure; the role of vitamin D in cardioprotection, pathogenesis of heart failure, as well as improved cardiac function in patients with heart failure remains to be fully established.

With the discovery of the presence of vitamin D receptor (VDR) in rat cardiomyocytes [5] more than 3 decades ago, a direct regulatory role of vitamin D3 or its active metabolite on cardiac contractility was also subsequently revealed. In this regard, vitamin D3 depletion in rats has been demonstrated to increase ventricular muscle mass as well as ventricular contractile function in the rat [6, 7, 8]. Indeed, animal studies have established that vitamin D deficiency is associated with heart failure risk factors including hypertension, cardiac hypertrophy and fibrosis [9]. Interestingly, the genetic disruption of the VDR has been reported to result in an overstimulation of the cardiac renin angiotensin system (RAS) leading to cardiac hypertrophy [10]. It was suggested that in VDR knock out mice that vitamin D regulates cardiac function through RAS. On the other hand, in the vitamin D deficient spontaneously hypertensive heart failure prone rat model, ventricular remodeling and the progression to the final terminal phase of heart failure phenotype was suggested to be associated with vitamin D deficiency and not with the initial cardiac hypertrophy [11]. The increase in LV diameter and cardiac output has been shown to be attenuated in 1,25-dihydroxyvitamin D3 treatment of spontaneously hypertensive heart failure prone (cp/+) rats and thus may prevent the development of cardiac hypertrophy and subsequent progression to heart failure [12]. Taken together, from the aforementioned, it would appear that pre-clinical studies have demonstrated that vitamin D has a protective action in the progression of cardiac hypertrophy and transition to heart failure.

The VDR has now also been reported to be expressed in human heart cells [13]. Vitamin D status is considered to be associated with the development and progression of human heart failure [14], and the prevention and correction of vitamin D deficiency may potentially reduce the incidence of heart failure [15]. Several clinical studies have reported a positive effect of vitamin D on heart function; however, there are other human studies that have reported opposing findings. Thus, it appears that there is a disagreement as to whether vitamin D supplementation is effective as a therapeutic agent for improved heart function in heart failure or if it is more effective as a component for the prevention of cardiac dysfunction in patients at risk for heart failure. In this article, we describe some of the mechanisms/sites of action of vitamin D in different animal models of heart failure, as well as review the role of vitamin D in human heart failure and discuss the current understanding and interpretation of data as well as clinical significance of insufficient vitamin D concentrations and of vitamin D supplementation on myocardial function in heart failure.

## 2. Metabolic Pathway and Role of Vitamin D

**Fig. 1.**
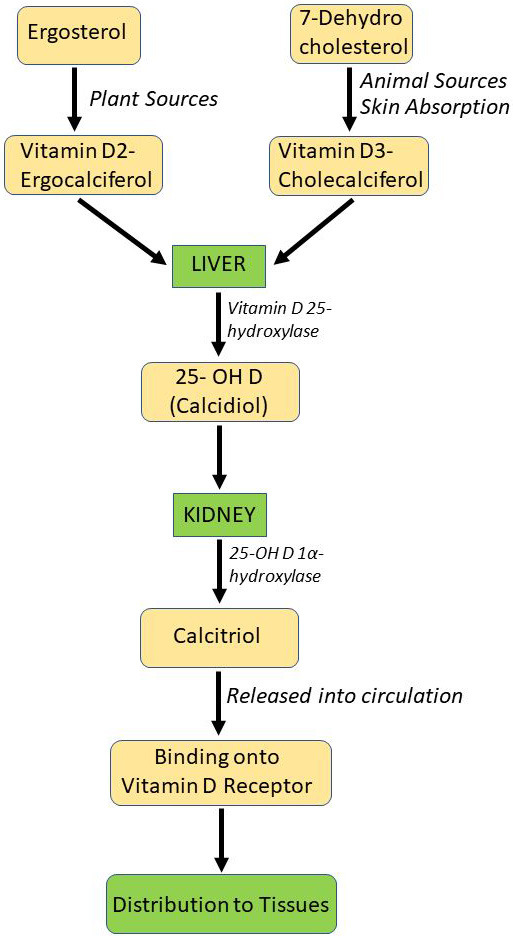
**The synthetic machinery and metabolic pathway of vitamin D**. The precursors of vitamin D synthesis (vitamin D2 ergocalciferol and vitamin D3-cholecalciferol) undergo 1st hydroxylation step in the liver, followed a 2nd hydroxylation of the formed calcidiol to produce the biologically active form of vitamin D- 1,25-OH2 vitamin D (calcitriol) for distribution to tissues via vitamin D binding protein. 25-OH D, 25-hydroxy vitamin D3.

The synthetic machinery and metabolic pathway of vitamin D is depicted in Fig. 1. There are two main forms of Vitamin D, ergocalciferol (vitamin D2), which is obtained from plant material or from dietary sources such as mushrooms [16]; and cholecalciferol (vitamin D3), which is formed in the skin after ultraviolet light exposure and thus, is typically synthesized during the summer months. However, it can also be obtained from nutritional sources such as fatty fish (salmon, tuna, and mackerel). Both vitamin D2 and vitamin D3 are hydroxylated and contribute to the main pool of 25-hydroxyvitamin D in blood serum. It should be noted that the recommendation of adequate sunlight exposure and dietary or supplemental vitamin D intakes are confusing for most people [17], because important modulators such as skin pigmentation, latitude of residence and season of the year are not taken into consideration.

The metabolic pathway of vitamin D involves two hydroxylation reactions in the body. The first, taking place in the liver, converts vitamin D to 25-hydroxyvitamin D (25-OH vitamin D), an intermediate metabolite. The second hydroxylation occurs in the kidney, where 25-OH vitamin D is converted to the dihydroxy form, 1,25-dihydroxyvitamin D (1,25-${\mathrm{OH}}_{2}$ vitamin D), the active metabolite of vitamin D. The 1,25-${\mathrm{OH}}_{2}$ vitamin D, a fat-soluble hormone, can enter cells and bind to the VDR activating calcium binding proteins that mediate calcium absorption in the gut [13]. The production of 1,25-${\mathrm{OH}}_{2}$ vitamin D is stimulated by parathyroid hormone (PTH) and decreased by excess levels of calcium [13]. Vitamin D receptors are ubiquitous throughout the body indicating that biological effects of vitamin D are robust and extensive [18].

The primary physiological role of vitamin D is in bone and mineral metabolism by promoting calcium absorption in the gut. However, besides its primary physiological role, in cases of vitamin D deficiency it is involved in several pathophysiological states including kidney disease, parathyroid dysfunction, sarcoidosis, rickets [13] and rheumatoid arthritis [19]. In recent years, scientific focus and clinical studies have gone beyond the known classical effects of vitamin D on skeletal health (osteoporosis, osteomalacia, etc.). Indeed, some studies have investigated the beneficial effects of vitamin D in different pathophysiological conditions including cardiovascular disease and diabetes as well as arterial stiffness, which is a predictor of cardiovascular events, metabolic syndrome, stroke and peripheral arterial disease [20]. However, some of the emerging data contradicts such findings [21], which are largely borne out from discrepancies regarding technical issues in the measurement of vitamin D and metabolites including a need for standardization of assay methods and consistency in the methods employed, inability to directly compare data obtained from different studies, a lack of agreement on the definition of deficiency/inadequate levels of vitamin and to confirm vitamin D status at baseline and prior to supplememtation [21]. There are also other important considerations that can contribute to inconsistent findings, which can affect circulating levels of vitamin D, for example, the presence of obesity, seasonal variation in relation to exposure to sunlight and dosing regimen and type of supplemnation used [21]. It is thus apparent that there is a need for harmonization of study results with respect to quantification of vitamin D, interpretation of data and clinical outcomes.

## 3. Cardiac and Subcellular Remodeling in Heart Failure

It is now well known that heart failure is invariably associated with cardiac hypertrophy and a compromised cardiac contractile function. The changes in the shape and size of cardiomyocytes, a process referred to as cardiac remodeling, leads to cardiac dysfunction in heart failure. In addition, experimental and clinical lines of evidence have also demonstrated defective functioning of different subcellular organelles, a process that has been referred to as subcellular remodeling [22, 23, 24]. Abnormal subcellular proteomic, molecular and structural changes have been attributed to prolonged hormonal imbalance, including the renin angiotensin system and the sympathetic nervous system, metabolic derangements, the occurrence of oxidative stress and development of ${\mathrm{Ca}}^{2+}$-handling malfunction [22, 23, 24, 25]. Given the multifunctionality of vitamin D, it is plausible that several of these elements may be the site of action for improved cardiac function in heart failure or in the prevention of cardiac dysfunction in patients at risk for heart failure such as diabetes, hypertension and obesity. The foregoing discussion will describe some of these mechanisms of action of vitamin D that have been identified mostly in pre-clinical investigations.

## 4. Proteomic and Molecular Mechanisms of Vitamin D Action

**Fig. 2.**
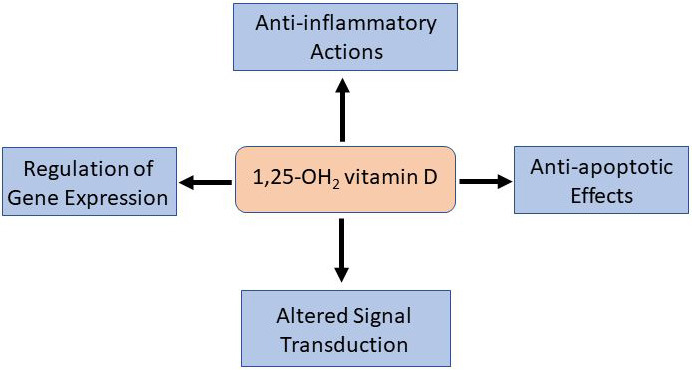
**Underlying mechanisms of action of vitamin D**. Experimental and some clinical studies have revealed several mechanisms of action for the potential benefits of vitamin D and include anti-inflammatory and anti-apoptotic effects as well as the ability to regulate gene expression and alter signal transduction processes.

A number of experimental investigations employing different animal models of heart failure have identified several different underlying mechanisms for the beneficial action of vitamin D and are summarized in Fig. 2. For example, some studies have revealed that vitamin D can regulate the processes involved in cardiac and extracellular matrix remodeling [17]. Vitamin D has also been reported to regulate both the renin angiotensin aldosterone system and the immune system [26, 27]. Indeed, vitamin D has been shown to down-regulate renin-angiotensin-aldosterone system hormones, and vitamin D3 repletion decreases aldosterone in patients with heart failure and low serum vitamin D [26].

*In-vitro* and animal models have demonstrated that vitamin D deficiency may have a contributory role in the inflammatory process, remodeling, fibrosis, and atherosclerosis in heart failure [28]. In a rat model of myocardial infarction induced by ligation of the left anterior descending coronary artery, proteins involved in energy metabolism, cardiac contractility, regulation of intracellular calcium, pathological hypertrophy and cardiac remodeling were shown to be differentially expressed [29]. With respect to inflammatory processes, a reduction in VDR expression, as well as increases in Th2 cells and Th2 cytokine production in myocarditis at end stage heart failure has been reported [30]; reconstitution of the vitamin D receptor in CD4+ T cells attenuated Th2-mediated inflammation. It was thus suggested that a deficiency in the VDR contributes to the development of myocarditis [30]. It should be mentioned that although an inverse correlation exists between 25-OH vitamin D and inflammatory markers, it is still contentious whether vitamin D lowers inflammation or whether inflammation lowers 25-OH vitamin D concentrations [31]. In this regard, it was suggested that both scenarios may be contributory factors [31]. While inflammatory process can be reduced by vitamin D, inflammation itself can hinder the metabolism of vitamin D resulting in an attenuation in 25-OH vitamin D levels; one does not excude or preclude the other and thus both these aspects may be important influences in the observed inverse relationship between 25-OH vitamin D and inflammation.

Interestingly, 1,25-${\mathrm{OH}}_{2}$ vitamin D has been shown to increase the expression of VDR in a dose-dependent manner [32]. Indeed, after an induced myocardial infarction in mice, treatment with 1,25-${\mathrm{OH}}_{2}$ vitamin D attenuated LV wall thinning and significantly improved LV systolic function [32]. It was suggested that the cardioprotective role of 1,25-${\mathrm{OH}}_{2}$ vitamin D was due to vitamin D mediated signal transduction and modulation of the cell cycle and regulation of stem/progenitor cell function [32]. Furthermore, a compound, referred to as VDR 4-1, has been revealed to exert strong transcriptional activities in a VDR reporter gene, and thereby attenuate cardiac hypertrophy, *in vitro* as well as in experimental models leading to restriction in the progression to heart failure [33].

In addition, paricalcitol, a synthetic analogue of vitamin D3 that selectively activates vitamin D receptors, has been reported to prevent the progression of ventricular dilation and hypertrophy as well as a reduction in ejection fraction, in a murine heart failure model of transverse aortic constriction [34]. It was found that amelioration of cardiac structure and function was related to the attenuation of the defects in intracellular ${\mathrm{Ca}}^{2+}$-handling, remodeling as well as anti-fibrotic and anti-hypertrophic actions. This analogue was also observed to exhibit anti-arrhythmic effects by preventing reductions in $K^{+}$-current density and the long QT, JT and TpTe intervals in heart failure animals [34]. Similarly, the improvement in cardiac function in response to paricalcitol treatment of mice following induction of myocardial infarction, has also been shown to be related to a reduction in apoptosis and inflammation [35]. Furthermore, VDR protein and mRNA levels were restored by paricalcitol treatment. Taken together, it is apparent that the cardioprotective effects of vitamin D subsequent to myocardial infarction can be attributed to an attenuation of the processes involved in inflammation, fibrosis, apoptosis, LV remodeling as well as anti-arrhythmic actions. Interestingly, the development of heart failure in Dahl salt-sensitive rats fed a high salt diet is prevented by paricalcitol in a mechanism involving a decrease in PKC α activation [36]. Indeed, a reduction in PKC α levels has been shown to be linked to an attenuation of the cardiac hypertrophic markers, brain natriuretic peptide (BNP) and atrial natriuretic factor (ANF) and improved cardiac function, in the same model, suggesting that vitamin D deficiency may be related to cardiac hypertrophy [37].

## 5. Role of Vitamin D in Human Heart Failure: Current Evidence and Controversies

The threshold values for vitamin D in the healthy population have been defined by two earlier seminal studies [38, 39]. In this regard, Sonderman *et al*. [39] have reported a sufficient serum level of 25-OH vitamin D as >20 ng/mL (50 nM), whereas a range of serum 25-OH vitamin D concentrations between 40–60 ng/mL (100–150 nM) has been reported as sufficient by Manson *et al*. [38]. This variation in the definition of optimal vitamin D status may confound the interpretation as well as the efficacy of vitamin D. Epidemiological data is suggestive of an association between low vitamin D and disease incidence. There are several epidemiological and clinical studies [40] that have reported a link between low vitamin D levels and different cardiovascular pathologies including coronary heart disease, heart failure and atrial fibrillation. However, results of interventional trials with vitamin D supplementation in patients at risk or with established cardiovascular disease (CVD) are contentious. Indeed, there is no clear scientific rationale for the use of vitamin D supplements in CVD [41].

In a post-hoc analysis conducted on the EVITA trial (Effect of Vitamin D on mortality in heart failure), the effect of 4000 IU daily vitamin D supplementation for up to 3 years on several cardiac functional and nephrological parameters including ejection fraction, left ventricular end-diastolic diameter (LVEDD) and left ventricular end-systolic diameter (LVESD) in patients with advanced heart failure was investigated [42]. Although no time and treatment interaction on these cardiac parameters were observed, an improved cardiac function as evidenced by a small but significant increase in LV ejection fraction was seen in patients aged 50 years or more. These investigators concluded that while vitamin D supplementation does not improve cardiac function in all advance heart failure patients, it did however, appear to improve LV function in older (≥50 years) patients [42].

Interestingly, the effects of a daily supplementation of 4000 IU vitamin D for 6 months in elderly patients with heart failure (mean age: 74 years) have also been reported to significantly increase LV ejection fraction. In addition, a decrease in systolic blood pressure was also observed in the intervention group. It should be mentioned that all patients in this study had vitamin D levels of <30 ng/mL which would be deemed as a deficiency state. However, supplementation significantly increases circulating levels of 25-OH Vitamin D [43].

Low plasma levels of 25-OH vitamin D are commonly observed in patients with advanced/chronic heart failure and have been linked to an increase in mortality risk [44, 45]. However, the EVITA 3-year randomized clinical trial also revealed that there was no reduction in the mortality rate in patients with advanced heart failure following a daily vitamin D dose of 4000 IU for 3 years, despite a normalization of plasma levels of 25-OH vitamin D in the supplemental group.

In a randomized, double-blind, placebo-controlled trial involving patients with stable New York Heart Association Class II-III heart failure and deficient or insufficient 25-OH vitamin D levels below 32 ng/mL, a 6-month intervention with daily vitamin D3 10,000 IU was associated with a repletion of 25-OH vitamin D and an improvement in quality of life (QOL) [46]. Of note, a normalization of BNP, PTH, and hs-CRP were also observed in the intervention group [46]. On the other hand, a 6-month randomized controlled trial of a 50,000 IU vitamin D3 weekly supplementation of patients with heart failure (mean age 65.9 years and mean ejection fraction of 37.6%) did not improve physical performance as evidenced by no changes in the peak VO2, 6-minute walk test (6-MWT) and knee isokinetic muscle strength even though there was a marked increase in 25-OH vitamin D [47]. Interestingly, this was a study where 48% of the patients were women and 64% African American which raises the possibility that sex and ethnicity may have some influence on outcomes in response to vitamin D supplementation. It should be noted that vitamin D supplementation has been suggested to be ineffective in being able to reduce cardiovascular risk factors (i.e., lipid profile and elevated blood pressure) in post-menopausal women with vitamin D deficiency [48].

Inconsistencies have been observed with respect to improvement in 6-MWT. In this regard, the ECSPLOIT-D study examined the effects of supplementation of vitamin D in patients with stable heart failure and a deficiency in serum levels of vitamin D of <20 ng/mL [49] . In this randomized, double-blind trial, the intervention group received 300,000 U loading dose of oral cholecalciferol followed by 50,000 U per month for 6 months. It was found that vitamin D supplementation in this patient population improved the 6-MWT, but only at 3 months of the supplementation period. Interestingly, an increase in left atrial size was observed in the placebo group [49]. In the VINDICATE study, which was a trial undertaken to investigate the effects of vitamin D on cardiac function in patients with chronic heart failure and vitamin D deficiency of <20 ng/mL [50], it was observed that supplementation with 4000 IU/day for 12 months did not improve 6-MWT. However, supplementation did significantly improve LV ejection fraction. In addition, it is notable that a reversal of LV remodeling as evidenced by a reduction in LVEDD and LVESD was also observed. It was concluded that while vitamin D supplementation does not improve the 6-MWT, it may exert beneficial effects on LV structure and function in patients with heart failure [50].

Information in the literature with respect to the effects of vitamin D supplementation on biomarkers as surrogates for the determination of heart disease are limited and inconclusive [51]. In this regard, the effects of monthly vitamin D supplementation at 100,000 IU on high sensitivity cardiac troponin I (hs-c Tn1), troponin T (hs-cTnT), and N-terminal-pro-B-type natriuretic peptide (NT-proBNP), as established biomarkers for heart failure in a post-hoc analysis have been examined. It was found that a reduction in plasma NT-proBNP levels occurred in those older adults with low Vitamin D status (i.e., <20 ng/mL) suggesting that there is a reduction in the risk of heart failure in this cohort. However, further work is required to demonstrate a causal relationship. Other potential mechanisms of vitamin D action have also been investigated. In a secondary analysis of the EVITA study of patients with advanced heart failure and serum 25-OH vitamin D concentration of <30 ng/mL who received 4000 IU of Vitamin D3 supplementation for 3 years, it was revealed that Vitamin D did not improve blood lipid parameters, i.e., total cholesterol, HDL cholesterol, LDL-cholesterol and triglycerides. Furthermore, there was no difference in the levels of vascular calcification inhibitor, fetuin-A [52]. Taken together, this analysis showed that vitamin D supplementation presents no benefit on the cardiovascular risk factors examined in this patient cohort.

It is pointed out that the low 25-OH vitamin D levels in patients with advanced heart failure is also associated with the occurrence of anemia [53]. A daily vitamin D supplementation of 4000 IU for 36 months did not reduce anemia prevalence in these patients. In addition, the progressive decline of renal function has been observed to be a frequent co-morbidity in patients with chronic heart failure [54]. In a study with a large cohort of patients with chronic heart failure, it was found that low 1,25-dihydroxyvitamin D/parathyroid hormone ratio is associated with an increase in the risk for deteriorating kidney function in patients with chronic heart failure. This ratio was also determined as an independent risk factor for hospital admission for cardiovascular events as well as for mortality [54]. Interestingly, low plasma vitamin D levels and the severity of the deficiency has been found to be an important predictor for in-hospital adverse cardiac events in patients hospitalized with first attack of acute myocardial infarction [55].

From the aforementioned, the findings of the VITAL heart failure study revealed that interventions with vitamin D do not significantly reduce the first heart failure hospitalization rate [56]. While the available data demonstrating beneficial therapeutic actions of vitamin D supplementation appear to be inconsistent, it is possible that maintenance of sufficient or adequate vitamin D levels may be preventive of heart disease. In this regard, a study conducted with healthy subjects aged 18–25 years with either insufficient (<20 ng/mL) or sufficient (>32 ng/mL) serum level of 25-OH vitamin D, the effects of daily vitamin D supplementation of 1200 IU on heart rate, systolic and diastolic blood pressures, as well as circulating norepinephrine levels were investigated [57]. Higher heart rate and higher systolic and diastolic pressures were observed in the vitamin D insufficient group, whereas serum norepinephrine levels were elevated in this group at baseline [57]. It was thus suggested that vitamin D may exert a modulatory action on the sympathetic nervous system and regulate norepinephrine levels in young adults.

The observation that no differences in these parameters occurred following longer intervention period is indicative that vitamin D may prevent heart disease in later life if sufficient amounts are maintained. It is interesting to note that more than half of the global population is estimated to be insufficient with respect to 25-OH vitamin D levels and thus strategies for the prevention of adverse health outcomes including heart disease should involve recommendations to take vitamin D supplements, particularly in winter months, or moderate exposure to sunlight, and to increase consumption of fish as well as of fortified foods [58].

It should be noted that the current recommendations for vitamin D supplementation assume that there are no differences in the requirements among ethnic or racial groups. Indeed, a study that examined vitamin D requirements among Caucasian and East African women residing in a Northern latitude, demonstrated that in order to maintain serum levels of 25-OH vitamin D of ≥12 ng/mL, it was estimated that more than a 2-fold higher intake of vitamin D was required in Somali women vs. Caucasian Finnish women. It was thus suggested that there are ethnic differences in the daily requirement of vitamin D and that it would be more appropriate to conduct dose-response studies based on ethnicity [59]. In this regard, despite high intakes of vitamin D as compared to Finnish counterparts, prevalence of vitamin D insufficiency has been reported among East African women living in Finland [60]. Furthermore, in the US 2006 NHANES survey of children, fewer than 1% of non-hispanic black children had optimal vitamin D status versus 25% of non-hispanic white children [61]. Indeed, ethnicity was considerably more significant than season or latitude and must be considered in recommendations for supplementation.

Overall, from the aforementioned clinical studies (summarized in the Table 1 (Ref. [42, 43, 44, 45, 46, 47, 48, 49, 50, 51, 52, 55, 56, 57]) below), the efficacy and response to vitamin D supplementation is dependent on several factors and the extrapolation of some clinical endpoints and surrogate markers of heart disease to a beneficial effect of vitamin D is not conclusive. Therefore, some reservation may be exercised in the recommendation of vitamin D supplementation in heart failure under a vitamin D insufficient state and thus more large-scale studies are warranted.

**Table 1.** **Clinical studies with vitamin D**.

Study population	Method of vitamin D supplementation	Outcomes	Reference
Advanced heart failure patients, 18–80 yrs old men and women	4000 IU oral D3 daily for 3 years	Increased LVEF in patients ≥50 years	[42]
		No reduction in mortality; associated with greater need for MCS implants	[45]
		No benefit on CVD risk factors	[52]
		Did not improve lipid profile and does not influence the calcification inhibitors fetuin-A and non-phosphorylated undercarboxylated MGP; no reduction in anemia	[55]
Chronic heart failure patients, mean age 74 yrs men and women	4000 IU D3 daily for 6 months	Increased/improved LVEF and lowered systolic blood pressure	[43]
Chronic heart failure patients, men and women	4000 IU D3 daily for 6 months	No improvement in endothelial function. Improvements in 6-minute walk distance, blood pressure, EuroQol 5D health questionnaire and left atrial diameter at 6 months	[44]
Class II/III NYHA men and women	10,000 IU oral D3 daily for 6 months	Improved QOL, normalized BNP, PTH and improved hsCRP in males	[46]
Heart failure patients, mean age 65, men and women	50,000 IU oral D3 weekly + calcium	No improvement in VO2, 6-MWT or knee isokinetic muscle strength	[47]
Postmenopausal women age 40–60; no CVD or diabetes	2000 IU oral D3 for 12 weeks	No effect on blood pressure or lipid profile	[48]
Heart failure patients	300,000 U oral D3 followed by 50,000 U monthly for 6 months	Improved 6-MWT but only at 3 months	[49]
Chronic heart failure patients, mean age 69 yrs men and women	4000 IU oral D3 daily for a year	No improvement on 6-MWD but has benefits on LV structure and function at 12 months	[50]
Mean age 66 yrs, men and women	100,000 IU oral D3 monthly for 1–2 years	Lower plasma NT-proBNP	[51]
Mean age 67 yrs, men and women	2000 IU oral D3 daily	No decrease in first hospitalization for heart failure rate	[56]
Healthy 18–25 yrs old either 25-OH vitamin D sufficient or insufficient men and women	1200 IU D3 daily	Higher HR, BP in vitamin D insufficient group	[57]

yrs, years; LVEF, left ventricle ejection fraction; MCS, mechanical circulatory support; CVD, cardiovascular disease; MGP, matrix Gla (γ-carboxylated glutamate); QOL, quality of life; BNP, brain natriuretic peptide; PTH, parathyroid hormone; hsCRP, high-sensitivity C-reactive protein; VO2, rate of oxygen; 6-MWT, 6-minute walk test; 6-MWD, 6-minute walk distance; NT-proBNP, N-terminal-pro B-type Natriuretic Peptide; HR, heart rate; BP, blood pressure.

## 6. Vitamin D in Diabetes, Obesity and Hypertension: Heart Failure Risk Factors

A link between deficient levels of vitamin D and chronic inflammatory diseases, such as diabetes and obesity, both of which are high risk factors for heart disease has been suggested, and for which, vitamin D supplements may exert a therapeutic benefit [62, 63]. However, the issue of low vitamin D status leading to type 2 diabetes is still unclear [64, 65]; in this regard, no association between vitamin D supplementation and prevention of type 2 diabetes was reported [64]. On the other hand, vitamin D supplementation has been shown to provide benefit in diabetes prevention if serum levels of 25-OH are <20 ng/mL, but not if serum vitamin D levels are >30 ng/mL [66]. Some other lines of evidence for the beneficial role of vitamin D in diabetes has been reviewed [67]. The data are suggestive that vitamin D may improve cardiac outcomes in diabetic patients through several different mechanisms including attenuation of inflammation, oxidative stress, cardiac remodeling, fibrosis, atherosclerosis as well as regulating advanced glycation end-product signaling [67]. Furthermore, 1,25-${\mathrm{OH}}_{2}$ vitamin D has been suggested to improve diabetic cardiomyopathy in type 1 diabetic rats by modulating autophagy through the β-catenin/TCF4/GSK-3β/mTOR pathway [68]. Although obesity has been linked to low vitamin D levels; vitamin D supplementation has been demonstrated not to induce weight loss and thus the association between vitamin D and obesity remains controversial [69].

The relationship between plasma fibroblast growth factor 23, PTH and 25-OH vitamin D with heart failure in a population-based study has been undertaken. It was found that an interaction between PTH and obesity was observed, suggesting a link with heart failure risk in obese individuals, although the role of PTH in the development of heart failure was unclear and there was no relationship between 25-OH vitamin D3 and heart failure [70]. Another study has recently examined the relationship between VDR genotypes, plasma concentrations of vitamin D metabolites and risk of heart failure and metabolic disorders (including obesity). It was determined that carriers of the TT ApaI, TC TaqI, and GA BsmI genotypes exhibited higher risk for obesity, whereas the FokI TT genotype was linked to an increase in the occurrence of heart failure and hypertension [71]. These investigators suggested that specific VDR genotypes are associated with circulating levels of 25-OH vitamin D. Taken together, low 25-OH vitamin D3 may be associated with a higher risk of diabetes and obesity and subsequent heart failure [72].

As already mentioned, vitamin D deficiency has been associated with hypertension and with seasonal variations in blood pressure and with the identification of vitamin D receptor and 1α-hydroxylase in endothelial and vascular smooth muscle cells, vitamin D has been implicated in the regulation of blood pressure [73, 74]. In fact, a deficiency in vitamin D in humans has been linked to the occurrence of hypertension, which may be related to the negative regulatory influence of vitamin D on the RAS [75], hyperactivity of which is known to regulate blood pressure. Interestingly, vitamin D levels have also been reported to be lower in patients with pulmonary hypertension [76, 77]. It should also be mentioned that an increase in pulmonary vascular resistance results in pulmonary hypertension leading to right heart failure and ultimately death. Although a deficiency in vitamin D can increase the predisposition to hypertension and LV dysfunction; the causative nature of low serum vitamin D concentrations and the incidence of pulmonary hypertension and right ventricular dysfunction is still unkonwn [78]. Overall, randomized trials have not demonstrated significant effects on CVD endpoints and therefore on current lines of evidence use of vitamin D supplements in vascular disease is not supported [74].

## 7. Consideration for the Type and Frequency of Vitamin D Supplement

It can be noted that all the aforementioned studies made use of cholecalciferol as the source of vitamin D supplementation. However, another commercially available source that can be used for supplementation is calcefidiol. Fig. 1 presents the metabolism of vitamin D upon oral administration. While both cholecalciferol and calcefidiol, the latter being a derivative of the former, are converted to calcifitriol prior to receptor binding and distribution into tissues, the two present a significant difference in pharmacokinetic profile particularly affecting absorption. A study comparing cholecalciferol and calcifediol supplementation showed that calcifediol caused a more rapid increase in serum 25-OH vitamin D levels and is more potent than cholecalciferol thereby requiring lower dosages [79]. In addition, calcifediol has a higher rate of intestinal absorption and exhibits a linear dose-response curve, thereby achieving an increase in 25-OH vitamin D levels dependent on dose and frequency of administration. This is in contrast with cholecalciferol which presents lower 25-OH vitamin D levels after administration due potentially to several factors such as obesity, liver failure, or severe intestinal malabsorption syndromes [79, 80]. Calcifediol’s high bioavailability can be attributed to its high affinity for the vitamin D-binding protein and its lower tendency to be trapped in adipose tissue [81].

Another study comparing the efficacy of weekly supplementation with either cholecalciferol or calcifediol in geriatric patients with hypovitaminosis D showed that both can effectively achieve optimum circulating levels of 25-OH vitamin D levels. However, the average value of 25-OH vitamin D in circulation was over 50% higher in patients receiving calcifediol compared to those receiving cholecalciferol [82]. Despite these evidences, the majority of clinicians still opt for cholecalciferol supplementation as it allows for a more varied frequency in administration which contributes to better treatment adherence [83]. This leaves calcifediol as an alternative form for patients with malabsorption problems. Regardless, taking into consideration both calcifediol and cholecalciferol as options for vitamin D supplementation may prove to be beneficial given diverse and unique patient factors.

The frequency of supplementation in the studies discussed ranged from daily to weekly to monthly. With the half-life of calcifidiol at approximately 15 days, consideration should be given to whether these differences may effect patient outcomes.

## 8. Technical Considerations When Measuring Vitamin D

There are a number of other aspects that should be considered when understanding the role of vitamin D deficiency as a causative factor for heart failure or as an effective therapeutic agent. In this regard, it is conceivable that low levels of vitamin D are secondary to heart failure as opposed to the primary cause of the disease. In addition, methodological concerns and the incorrect measurement of serum levels of 25-OH vitamin D may be providing a false-positive regarding associations between low vitamin D and heart failure. There are also number of factors that influence/modulate circulating levels of vitamin D including, different geographic latitudes, skin pigmentation, availability of vitamin D food sources, age, sex, cultural habits and lifestyle [17]. Furthermore, hypoparathyroidism, severe kidney disease and liver insufficiency will affect serum 25-OH vitamin D levels [84]. Excessive intake of vitamin D has been shown to cause vitamin D toxicity that can lead to anorexia, weight loss, polyuria, and heart beat irregularities. Vitamin D toxicity can also lead to elevated calcium, which can then cause calcification of vasculature and tissues with subsequent damage to internal organs such as the heart and kidneys [85]. Altogether, these reports have led to confusion regarding recommended levels of vitamin D for healthy individuals vs. those with various disease states [21].

The major contributing factor that leads to such controversy is that the current optimal serum vitamin D level has yet to be established. Furthermore, despite the large amount of data, the definition of the optimal status of vitamin D still remains to be defined. Indeed, there is still a lack of consensus on threshold values, the consequences of inadequate or insufficient levels of vitamin D, the daily intake needed, and toxicity of vitamin D [17, 86, 87, 88]. Several factors including; metabolite species, technique, methodology, and analysis are not standardized [21]. Large variability exists among assays (i.e. competitive immunoassay vs. liquid chromatography followed by mass spectroscopy) and laboratories [89, 90]. The current accepted method for determining vitamin D status is by measuring serum concentration of 25-OH vitamin D because this metabolite has a long half-life (~15 days) [91]. However, since vitamin D is stored in fat tissue, serum levels of 25-OH vitamin D do not correlate well with health status. Moreover, measurement of 25-OH vitamin D does not predict conversion to the active metabolite 1,25-${\mathrm{OH}}_{2}$ vitamin D. 1,25-${\mathrm{OH}}_{2}$ vitamin D has a shorter half-life of ~15 hours [91] and is tightly regulated by PTH, calcium, and phosphate. Therefore, it is not a good single indicator of Vitamin D deficiency since the level of 1,25-${\mathrm{OH}}_{2}$ vitamin D would not reflect vitamin D deficiency unless it was severe. Also, it should be mentioned that several different stability/storage/transport issues may result in inaccurate testing of 25-OH vitamin D [92]. Thus, in addition to the aforementioned controversies regarding vitamin D status, supplementation and heart failure, inaccurate and inappropriate measurement of vitamin D status may also contribute to the uncertainties.

## 9. Conclusions and Recommendations

A deficiency of vitamin D has been linked to the etiology and development and progression of heart failure; however, the role of vitamin D in human heart failure is still uncertain. Although epidemiological data have been confirmed by some experimental data, which show that knockout mice for the VDR developed myocardial hypertrophy and dysfunction, there is still substantial discrepancy between the outcome of experimental studies and clinical intervention trials. Thus, more research is needed to confirm whether add-on supplementation therapy with vitamin D has a role in the management of patients with chronic heart failure [93]. Indeed, recent clinical intervention studies have not shown a causal relationship between vitamin D supplementation and cardioprotection.

Several mechanisms for the beneficial effects have been proposed and are attributed to the experimental findings that demonstrate anti-inflammatory, anti-apoptotic, anti-fibrotic actions as well as protection from cardiac and subcellular remodeling. In view of the current pool of ambiguous evidence [94], some of the inconsistencies in the findings appear to be related to age, sex, ethnicity, geographic location and the metabolic pathway for the generation of the active dihydroxylated form of vitamin D. Furthermore, it is plausible that some of the controversies associated with the role of vitamin D in heart failure could be borne out from technical aspects particularly related to the measurement of vitamin D as well as the definition of optimal vitamin D status. It is important to note that current recommendations for vitamin D do not take some of these factors into consideration. Overall, it can be suggested that representative studies across different countries with highly comparable patient populations and analytical techniques need to be conducted to address these inconsistencies. While evidence supporting the therapeutic use of vitamin D supplements is inconclusive with respect to heart failure, supplementation to maintain adequate vitamin D levels may be of value as a preventive strategy.
